# Determination of bacteriocin activity with bioassays carried out on solid and liquid substrates: assessing the factor "indicator microorganism"

**DOI:** 10.1186/1475-2859-5-30

**Published:** 2006-10-10

**Authors:** Maria Papagianni, Nicholaos Avramidis, George Filioussis, Despina Dasiou, Ioannis Ambrosiadis

**Affiliations:** 1 Department of Hygiene and Technology of Food of Animal Origin, Laboratory of Food Technology, School of Veterinary Medicine, Aristotle University of Thessaloniki. Thessaloniki 54006, Greece; 2 School of Mathematics, Faculty of Sciences, Aristotle University of Thessaloniki. Thessaloniki 54006, Greece

## Abstract

**Background:**

Successful application of growth inhibition techniques for quantitative determination of bacteriocins relies on the sensitivity of the applied indicator microorganism to the bacteriocin to which is exposed. However, information on indicator microorganisms' performance and comparisons in bacteriocin determination with bioassays is almost non-existing in the literature. The aim of the present work was to evaluate the parameter "indicator microorganism" in bioassays carried out on solid -agar diffusion assay- and liquid -turbidometric assay- substrates, applied in the quantification of the most studied bacteriocin nisin.

**Results:**

The performance of characterized microorganisms of known sources, belonging to the genera of *Lactobacillus*, *Pediococcus*, *Micrococcus *and *Leuconostoc*, has been assessed in this work in the assays of plate agar diffusion and turbidometry. Dose responses and sensitivities were examined and compared over a range of assay variables in standard bacteriocin solutions, fermentation broth filtrates and processed food samples. Measurements on inhibition zones produced on agar plates were made by means of digital image analysis. The data produced were analyzed statistically using the ANOVA technique and pairwise comparisons tests. Sensitivity limits and linearity of responses to bacteriocin varied significantly among different test-microorganisms in both applied methods, the lower sensitivity limits depending on both the test-microorganism and the applied method. In both methods, however, only two of the nine tested microorganisms (*Lactobacillus curvatus *ATCC 51436 and *Pediococcus acidilactici *ATCC 25740) were sensitive to very low concentrations of the bacteriocin and produced a linear-type of response in all kinds of samples used in this work. In all cases, very low bacteriocin concentrations, e.g. 1 IU/ml nisin, were more accurately determined in the turbidometric assay.

**Conclusion:**

The present work shows that in growth inhibition techniques used in bacteriocin quantification, the choice of the indicator microorganism is critical. Evaluation of sensitivity levels and type of produced responses showed that they can vary widely among different test-microorganisms and different applied methods, indicating that not all microorganisms can be used successfully as indicators and that measurements of growth inhibition in liquid media produce more reliable results.

## Background

Bacteriocins are antimicrobial peptides or proteins produced by lactic acid bacteria (LAB). Their potential applications in the food and health care sectors [1] have attracted the strong interest of academia and the industry resulting in an impressive amount of published research on bacteriocin production, purification, genetics, and applications. So far, only nisin, the most studied bacteriocin produced by some strains of *Lactococcus lactis*, is produced commercially following designation as GRAS substance in the USA and specific approval in the EU. Today, nisin is an approved food additive in most major food producing countries. Another well-studied bacteriocin that will likely be the next to be used in the food industry is pediocin [2-4].

A major difficulty in bacteriocin research and applications is obtaining accurate quantification using bioassays which are based on the quantification of the inhibition produced in a sensitive microorganism [4-7]. These type of assays are the most widely used techniques for quantitative determination of bacteriocins. Although numerous other methods have also been described such as ELISA [8,9], ATP-bioluminometry [10], radiometry [11], conductance measurements [12], or even sophisticated bioassays based on self-induction of the *nis *promoter and bioluminescence [13], they have not gained wide acceptance because of requirements for dedicated equipment, supplies and skills, and more over because the results produced by such methods cannot necessarily be correlated with antimicrobial activity [4,14]. Therefore, growth inhibition techniques are still the most commonly used in everyday trials.

Multiple procedures based on growth inhibition are described in the literature, relying on tests performed either in solid, e.g. the plate agar diffusion assay, or in liquid medium, e.g. turbidometry. The agar diffusion assay [15-17], in which inhibition zones are produced in plates in a procedure similar to that of antibiograms, is undoubtedly the most commonly used despite the inconveniences and limitations of its application [18]. The performance of the method, which is laborious and time-consuming, depends largely on human ability and judgment and precision cannot be obtained when inhibition zones are unclear or not perfectly circular. Diffusion-related difficulties of the active substance represent another important limitation of agar diffusion assays.

The need to eliminate diffusion-related problems associated with the agar techniques, introduced liquid medium methods, which make use of indicator organisms and quantify the bacteriocin concentration from the percentage of growth inhibition in the indicator organism. The method was introduced by Reeves [19] in a study with colicins. Since then, applications of turbidometric assays can be found in a number of reports [20-22] in which, as with the agar diffusion assay, various indicator microorganisms were used, in procedures which show large variability regarding bacteriocin extraction, general experimental conditions and definition of the bacteriocin unit.

Successful application of growth inhibition techniques obviously relies on the sensitivity of the chosen indicator microorganism to the bacteriocin to which is exposed. Modifications of the first version of the agar diffusion assay [15], proposed by Rogers and Montville [16] and Wolf and Gibbons [17], introduced different microorganisms as better performing nisin-indicators and argued over the necessity of a pre-incubation period and the use of various diffusion-aiding surfactants (Tween solutions). Apart from published papers, a large number of versions of the agar diffusion assay are available in the web in the form of teaching material in laboratory courses from universities throughout the world. In each one of these versions, a different species of bacteria is proposed for use as indicator microorganism. To the best of our knowledge, there is no published report on indicator microorganisms' performance comparisons in bacteriocin determination with bioassays.

The aim of the present work was to evaluate the parameter "indicator microorganism" in the agar diffusion and turbidometric assays applied in the quantification of the most studied bacteriocin, nisin. The response of a test-microorganism to nisin is strain-dependent and responses of different strains within the same species could vary widely. This could be a serious problem in attempting to follow a published method that employs a not-widely available strain. Known strains-easily available- were employed, belonging to the genera of *Lactobacillus*, *Leuconostoc*, *Micrococcus*, and *Pediococcus *and their sensitivity was evaluated within a broad range of nisin concentrations in standard solutions, fermentation broths' filtrates and processed food samples, in both assays. Assay variables, like growth conditions, incubation periods and addition of surfactants, were also preliminary examined and evaluated for each tested microorganism. To eliminate the human judgment factor, inhibition zones were measured with an automated image analysis system.

## Results and discussion

Nisin can control a range of bacteria belonging to the following genera [23]: *Alicyclobacillus*, *Bacillus*, *Clostridium*, *Desulfotomaculum*, *Enterococcus*, *Lactobacillus*, *Leuconostoc*, *Listeria*, *Micrococcus*, *Pediococcus*, and *Sporolactobacilllus*. Nisin does not control Gram-negative bacteria, yeasts and moulds. From the sensitive to nisin bacteria, those characterized as non-pathogenic, belonging to "biosafety level 1", could be tested as potent indicators in a routine bioassay. The selected 9 strains, listed in the Materials and Methods section, belong to all genera of bacteria with the required characteristics and they can be screened for their dose-responses and sensitivity to nisin.

In both bioassays, the physiological state of the applied test-microorganisms corresponded to the middle of their logarithmic phase of growth. This was obtained at different time-points. Table 1 shows the values of OD_650 _and culture pH at the middle of the log phase (in parenthesis the corresponding time), as well as values of OD_650_, colony forming units (cfu) and cell dry-weighs (CDW) at 24 hours of culture of the test-microorganisms used. *L. plantarum *showed better growth compared to others, with 3.25 g/l biomass concentration, followed by the two *Pediococcus *strains with 2.12 and 2.62 g/l biomass concentration.

**Table 1:** Culture characteristics of test-microorganisms used in agar diffusion and turbidometric assays for determination of nisin

**Microorganism**	**OD**_**650 **_mid-log phase	**pH **mid-logphase	**OD**_**650 **_24-hrs	**CDW (g/l) **24-hrs	**cfu/ml **24-hrs
*L. plantarum*	0.355	6.35	1,114	3,250	2,9 × 10^9^
*L. curvatus*	0.347	6.35	0,933	1,500	8,5 × 10^9^
*L. sakei*	0.345	5.95	0,849	1,400	2,7 × 10^9^
*M. luteus*	0.362	5.95	1,041	1,920	2,5 × 10^9^
*M. varians*	0.303	5.80	1,001	1,800	2,6 × 10^9^
*M. flavus*	0.362	5.80	1,054	1,750	2,6 × 10^9^
*L. mesenderoides*	0.350	5.90	0,934	1,450	7,0 × 10^9^
*P. acidilactici*	0.313	6.00	1,057	2,120	2,6 × 10^9^
*P. pentosaceus*	0.340	6.00	1,001	2,620	9,6 × 10^8^

### Agar diffusion assay

Diameters of inhibition zones produced in plates by the various test-microorganisms exposed to equal nisin concentrations showed wide variation. The data collected were examined with the ANOVA technique to evaluate the effects of the factors X1 – nisin concentration level (dose) and X2 – microorganism, as well as their interdependence X1*X2, on the variable Y1 – diameter of inhibition zone. The analysis of variance for the agar diffusion assay is presented in Table 2. The factors X1, X2 and X1*X2 had a significant effect on the variable Y1 and therefore on prediction of its values. Value differences for each factor were examined in pairwise comparisons tests and showed that for both X1 and X2, differences were statistically important at the significance level of α = 0,05.

**Table 2:** AOV Table for evaluation of the effects of nisin concentration and microorganism employed in assay and their interdependence on the agar diffusion and turbidometric assays

***Source***	***Type III Sum of Squares***	***df***	***Mean Square***	***F***	***Sig***.
***Agar Diffusion Assay***					
Dependent variable Y1					

Model	7101,610^a^	90	78,907	3219,225	,000
X1	3034,214	14	216,730	8842,095	,000
X2	716,658	5	143,332	5847,619	,000
X1*X2	283,388	70	4,048	165,166	,000
Error	11,030	450	2,451E-02		
Total	7112,640	540			
^a ^R squared = ,998 (Adjusted R Squared = ,998)					

***Turbidometric Assay***					
Dependent variable Y2					

Model	2544723,345^a^	90	28274,704	10586,138	,000
X1	399015,846	14	28501,132	10670,913	,000
X2	365947,762	5	73189,552	27402,398	,000
X1*X2	129160,436	70	1845,149	690,830	,000
Error	1201,913	450	2,671		
Total	2545925,258	540			
^a ^R squared = 1,000 (Adjusted R Squared = ,999)					

**Figure 1:**
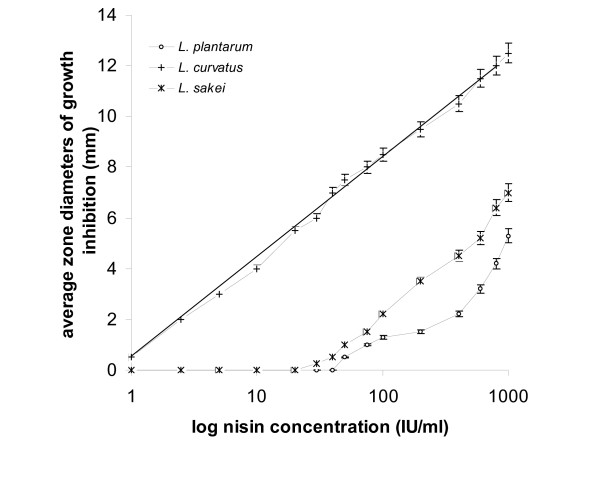
*L. plantarum*, *L. curvatus *and *L. sakei *in agar diffusion assay: plots of log_10 _nisin concentrations *vs *average zone diameters of growth inhibition.

**Figure 2:**
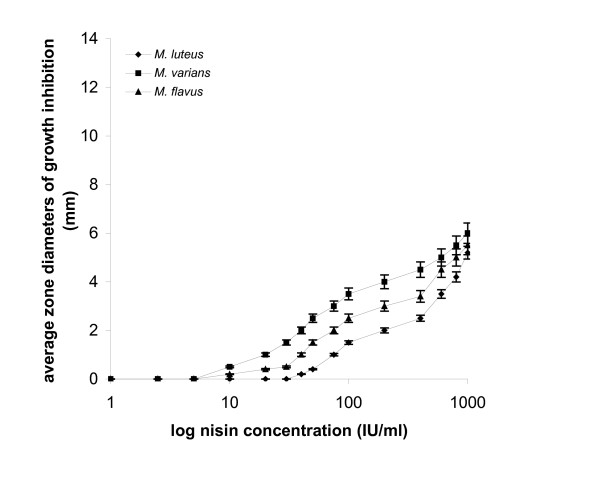
*Micrococcus *spp. in agar diffusion assay: plots of log_10 _nisin concentrations *vs *average zone diameters of growth inhibition.

**Figure 3:**
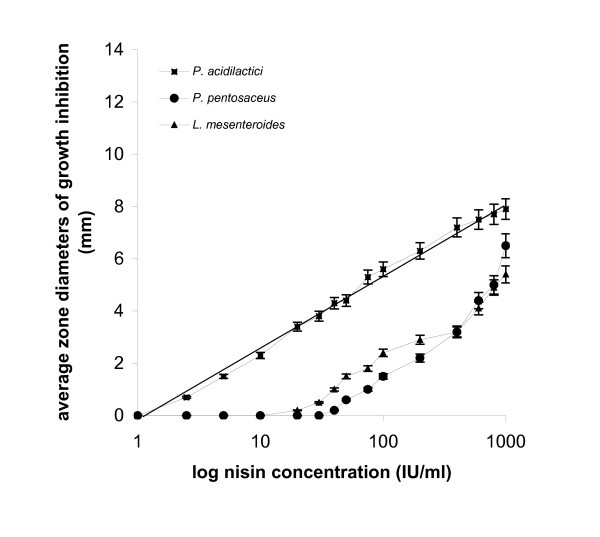
*P. acidilactici*, *P. pentosaceus *and *L. mesenteroides *in agar diffusion assay: plots of log_10 _nisin concentrations *vs *average zone diameters of growth inhibition.

Log nisin concentrations in the range of 1 to 1.000 IU/ml were plotted against average diameters of inhibition zones of inhibition as shown in Figs 1, 2, and 3. According to Fig. 1, from the three *Lactobacilli *tested, *L. curvatus *produced a linear response having an *R *value greater than 0.98. *L. curvatus*, appeared to be the most sensitive to nisin, producing the largest zones, with 12 mm corresponding to the dose of 1.000 IU/ml nisin, and a minimal detectable level of 1 IU/ml. A linear response was also observed with *L. sakei *to nisin levels between 40 and 1.000 IU/ml. However, *L. sakei *sensitivity was by far lower compared to the exhibited by *L. curvatus*, since no response was observed to doses up to 40 IU/ml nisin. *L. plantarum *produced inhibition zones at nisin concentrations above 75 IU/ml, while its response appeared sigmoidal. *L. sakei *and *L. plantarum *produced significantly smaller inhibition zones compared to those produced by *L. curvatus*, ranging from 0.25 to 7 mm and 0.5 to 5.3 mm, respectively, for nisin concentrations ranging from 40 to 1.000 IU/ml.

Responses from the three *Micrococci *(Fig. 2) were obtained at nisin concentrations exceeding 40 IU/ml and produced sigmoidal plots. Inhibition zones were of small diameter, ranging from 0.2 to 5.8 mm for 40 to 1.000 IU/ml nisin. *L. mesenteroides *sbs. *cremoris *appeared insensitive to doses lower than 75 IU/ml nisin (Fig. 3) and the diameters of the produced zones ranged from 0.2 to 5.2 mm.

*P. acidilactici *appeared to be sensitive to concentrations as low as 2.5 IU/ml nisin (Fig. 3), producing large inhibition zones with an average diameter approximating 7.9 mm at 1.000 IU/ml nisin, and a linear response with the *R *value equal to 0.98. As also shown in Fig. 3, *P. pentosaceus *produced inhibition zones at nisin levels above 40 IU/ml and the diameter of zones reached a maximum of 6.5 mm at 1.000 IU/ml nisin.

In plate assays, performed in solid substrate, a linear relationship between the logarithm of the dose and the inhibition is generally accepted, the latter being normally estimated as the diameter of the zone. In many cases, as with *L. plantarum *in this study, the response is far from linear (Fig. 1). Agar diffusion assays data show that the performance of test-microorganisms varies in terms of sensitivity and linearity of response and therefore, not all of them can be used safely in bacteriocin activity determination. Linear responses obtained with: *L. curvatus *for nisin concentrations throughout the range of 1 to 1.000 IU/ml, the inhibition zone diameters ranging from 0.5 to 12.5 mm; *P. acidilactici*, within the range of 2.5 to 1.000 IU/ml nisin, producing zones of a diameter ranging from 0.7 to 7.9 mm; and *M. varians*, within the range of 10 to 1.000 IU/ml nisin, producing zones of a diameter ranging from 0.5 to 6 mm.

### Turbidometric assay

Quantitative activities of nisin in standard solutions were determined in liquid substrate with turbidometry, using the same test-microorganisms as with the agar diffusion assay.

Mean values of 3 independent measurements of OD_650 _were used to estimate the percentage of growth inhibition against the controls. As with the agar diffusion assay, the effects of the factors X1 -nisin concentration level (dose), X2 -microorganism employed, and their interdependence X1*X2, on the variable Y2 – % inhibition of growth were examined with the ANOVA technique for the turbidometric assay. The analysis of variance for the turbidometric assay is presented in Table 2. The factors X1, X2, and X1*X2 had a significant effect on the variable Y2 and on prediction of its values. Value differences for each factor were examined in pairwise comparisons tests and showed that for both factors X1 and X2, differences were statistically important at a significance level of α = 0,05.

**Figure 4:**
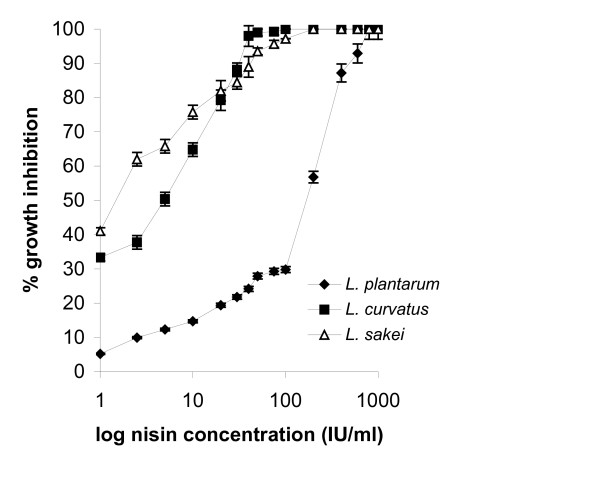
Turbidometric assay: plot of log_10 _nisin concentrations vs percentage inhibition of growth with *L. plantarum*, *L. curvatus *and *L. sakei*.

**Figure 5:**
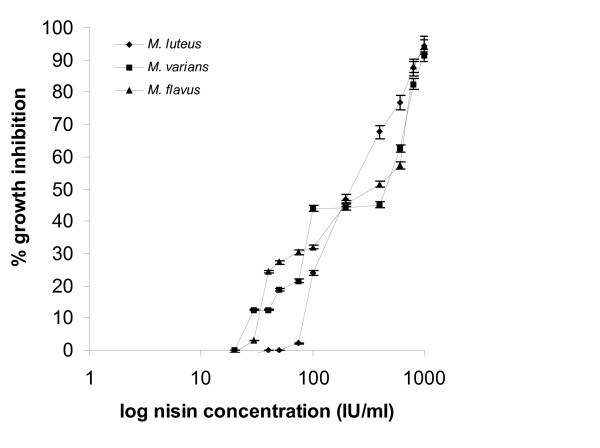
log_10 _nisin concentrations vs percentage inhibition of growth by *Micrococcus *spp. in the turbidometric assay.

**Figure 6:**
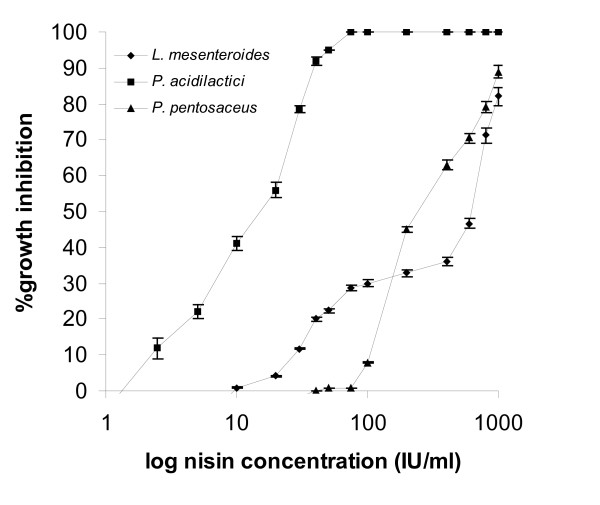
Turbidometric assay: plot of log_10 _nisin concentrations vs percentage inhibition of growth with *P. acidilactici*, *P. pentosaceus *and *L. mesenteroides*.

Percentages of growth inhibition of test-microorganisms were plotted against log nisin concentrations in Figs 4, 5, and 6. Total inhibition was observed with *L. curvatus *at 75 IU/ml nisin, with *L. sakei *at 100 IU/ml, while with *L. plantarum *only at 800 IU/ml nisin. The lower response limit for all three *Lactobacilli *was 1 IU/ml nisin, at which growth inhibition was 33% for *L. curvatus*, 41% for *L. sakei*, and 5.20% for *L. plantarum*. This method is obviously more sensitive than the agar diffusion method since lower detected sensitivity levels in the agar were 40 IU/ml for *L. sakei *and 75 IU/ml for *L. plantarum*. *L. plantarum *appeared to be less sensitive to nisin compared to the other two *Lactobacillus *strains, but it produced a linear response to nisin doses ranging from 1 to 100 IU/ml. *L. curvatus *responded linearly within 1 to 75 IU/ml nisin (*R *> 0.98) -the response producing a plateau at higher nisin concentrations- suffering a much stronger inhibition effect. The results produced with *L. curvatus *in the turbidometric assay are in agreement with those produced in the agar diffusion assay. *L. sakei *response, according to Fig. 5, cannot be regarded as linear.

Compared to *Lactobacillus *strains, *Micrococcus *strains appeared to be less sensitive to nisin (Fig. 5) since the maximum growth inhibition effect was obtained at 1.000 IU/ml nisin and approximated a 90%. *M. varians *and *M. flavus *responses commenced at 30 IU/ml nisin, with 12% and 3% inhibition of growth, respectively, while *M. luteus *response commenced at 75 IU/ml nisin with a 2% inhibition of growth. Plots of percentages of growth inhibition against log nisin concentrations produced sigmoidal type curves for all three *Micrococcus *strains and this is in agreement with the results obtained with the agar diffusion assay. *L. mesenteroides *sbs. *cremoris *response to nisin levels commenced at 10 IU/ml and its maximum inhibition of growth was 82% at 1.000 IU/ml nisin.

From the two *Pediococci *tested, *P. acidilactici *produced a strong response with a lower detection limit for nisin at 2.5 IU/ml with 12% inhibition of growth, and 100% inhibition of growth at 75 IU/ml. The response of *P. acidilactici *was linear for doses of 2.5 to 75 IU/ml nisin. *P. pentosaceus *was sensitive to nisin concentrations above 40 IU/ml and its maximum growth inhibition at 1.000 IU/ml was approximately 90%. The two *Pediococci *therefore, produced comparable results in the two bioassays.

Concluding on the performance of tested microorganisms in the turbidometric assay, *L. curvatus *appeared to be the most sensitive, producing a linear response, with 33% inhibition at 1 IU/ml and 100% inhibition at 75 IU/ml nisin. Next to *L. curvatus, P. acidilactici *appeared also to be very sensitive, producing a linear response to nisin, with initial response at 2.5 IU/ml nisin with 12% growth inhibition and 100% growth inhibition at 75 IU/ml nisin. It should be noted however here that absence of lineariry is rather common in turbidometric assays, as it appears in the studies of Berridge and Barrett [24], Shannon and Hedges [25], and Parente et al. [26]. Interpolation was carried out in most of these cases, either under the supposition that linearity exists over the whole response interval [27], or that the relationship between dose and the proportion of inhibition is taken to be linear when it lies between 20 and 80% [19]. Comparing the performances of the tested microorganisms in the two bioassays, it appears that *L curvatus *ATCC 51436 and *P. acidilactici *ATCC 25740 produce the kind of response to nisin which makes them suitable for use as indicator microorganisms, the first being by far the most sensitive. Our results show that in bacteriocin determination with a microbiological method, the choice of the indicator microorganism is critical. However, the plate agar diffusion method itself has its well-known limitations. Low active nisin concentrations can be safely determined only by the turbidometric assay. The use of image analysis in this work, permitted measurements of otherwise unclear zones of very small diameters and proved valuable in determining the sensitivity of test-microorganisms at very low nisin levels. It is very characteristic that the response of *L. curvatus *to 1 IU/ml nisin was a zone of an average diameter 0.5 mm in the agar diffusion assay, while a 33% inhibition of growth in the turbidometric assay.

### Determination of bacteriocin activity in culture broth filtates

**Figure 7:**
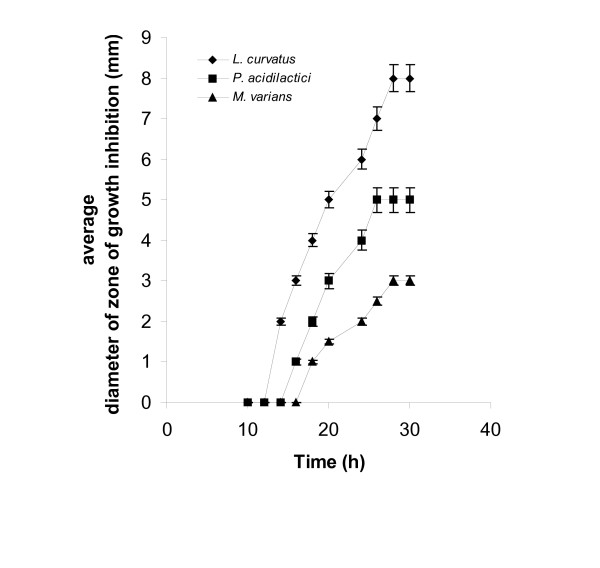
Determination of nisin in *L. lactis *fermentation broths with the agar diffusion assay using *L. curvatus*, *P. acidilactici*, and *M. varians*.

**Figure 8:**
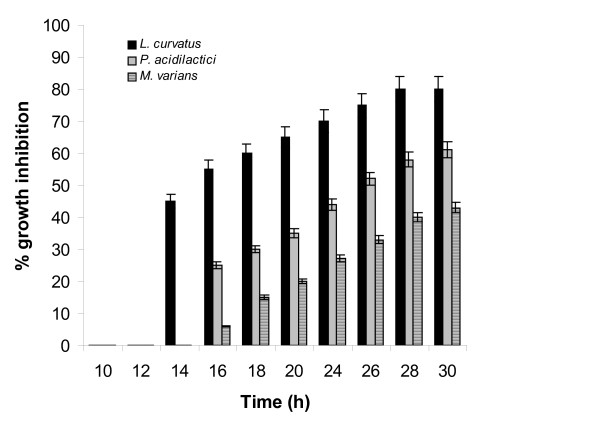
Turbidometric assay: determination of nisin in *L. lactis *fermentation broths using *L. curvatus*, *P. acidilactici*, and *M. varians*.

Following treatment, samples were analyzed for nisin with both methods and all test-microorganisms. *L. curvatus *and *P. acidilactici *were the test-microorganisms performing better- in terms of produced zones of inhibition and percentages of growth inhibition- in both assays than the other examined microorganisms. Figures 7 and 8 show the results produced with the agar diffusion and the turbidometric assays, for *L. lactis *fermentation samples, using *L. curvatus*, *P. acidilactici *and *M. varians*. Nisin concentrations in *L. lactis *fermentation broths can be quantified successfully using *L. curvatus *as an indicator microorganism using the standard curves produced with standard nisin solutions, made with nisin from Sigma, in the agar diffusion and the turbidometric assays.

### Determination of bacteriocin activity in food samples

Microbiological assays of nisin in foods are not specific since other antibiotics present in food could interfere. In preparation for nisin assay, samples of food were acidified and boiled in order to bring nisin into the aqueous phase, while in a second stage, the nisin-containing extracts were made alkaline and heated to inactivate nisin and provide a nisin-free diluent for standard. The fate of other antibiotics when subjected to heat under acid and alkaline conditions is an important factor when considering the possibility of interference during nisin assay. According to data reported by FAO [28] it appears that two antibiotics, namely tylosin and polymyxin B, might be confused with nisin when measuring antibiotic activity in foods.

Food extracts, prepared as described in the Materials and method*s *section, were tested for nisin with the agar diffusion assay without prior inactivation of nisin, following treatment for inactivation of nisin, and after addition of known nisin concentrations to the treated for nisin inactivation samples. Table 3 shows the results from agar diffusion assays carried out with the same dilutions of supernatants and same added nisin concentrations using *L. curvatus*. It is obvious that the inhibition zones produced on agar plates in the case of shrimp paste refer to the presence of antimicrobial substances that remained active after the nisin inactivation treatment. Characteristic is that the same amount of added nisin produced larger zones of inhibition in the presence of extract of the shrimp paste than in 0.02 N HCl. Plots of the results produced lines of a very different slope, a strong indication that the level of interfering substances is rather significant (results not shown). Samples from the cream and chicken soup and cream cheese extracts produced inhibition zones before treatment but not following the nisin inactivation treatment. Nisin was added by the manufacturer to those products, (according to package information), and as none of the controls in the case of these two products produced an inhibition zone, it can be concluded that the extracts were satisfactorily freed from nisin by the alkali treatment.

**Table 3:** Determining nisin in food extracts with the agar diffusion assay and the indicator microorganism *L. curvatus*. Extract treatment (under the same dilution factor and addition of the same concentration of nisin) and inhibition zones formation

Source of Extract	Zones of inhibition			
Treatment	Untreated	Alkali treated	Nisin added	Control

Cream of chicken soup	+	-	+	-
Shrimp and cheese paste	+	+	+	-
Mayonnaise	-	-	+	-
Marinated herring	-	-	+	-
Cream cheese	+	-	+	-

A range of known nisin concentrations were added to the alkali treated food extracts and examined for production of growth inhibition zones with *L. curvatus *ATCC 51436 and *P. acidilactici *ATCC 25740. The reference concentrations and unknowns were delivered, randomized, in quadruplicate into the holes on assay plates. The average zone diameters for the reference concentrations were plotted against log nisin concentrations and the nisin content of the unknowns was read off from the straight line produced. The results showed good agreement in the nisin contents obtained from dilutions of the same food extract and bear out the results for use of alkali-treated extracts as diluents for the reference nisin concentrations. *L. curvatus *appeared again to be more sensitive in low nisin concentrations detecting nisin to the 1 IU/ml level and producing a linear response. However, the average zone size produced by standard nisin solutions and alkali-treated food samples was not the same. Larger zones compared to those produced by standards, were produced in the case of alkali-treated food extracts to which nisin was added and this obviously refers to substances interfering the diffusion process. Since the antibiotic-free extracts examined never produced any zones of their own, this is due to the presence of substances capable of behaving as surface-active agents enhancing the diffusion of nisin into the agar. It appears that in the estimation of nisin in food extracts, controls consisting of standard nisin solutions, as well as of nisin-free extracts into which nisin is incorporated are absolutely necessary. This procedure will compensate for the effect on zone size of factors present in food extracts.

**Figure 9:**
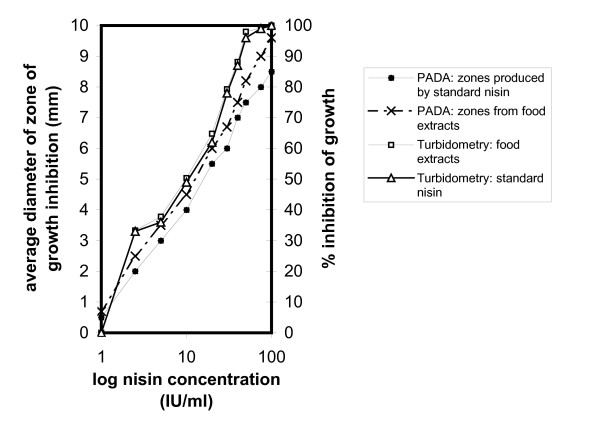
Comparisons of performances of *L. curvatus *in the plate agar diffusion (PADA) and the turbidometric assays in determination of nisin in processed food extracts and standard nisin solution. Plots of log_10 _nisin concentrations *vs *average zone diameters of growth inhibition.

Nisin estimation in food extracts and controls treated as described above, was done with the turbidometric assay and the response of the same indicators *L. curvatus *ATCC 51436 and *P. acidilactici *ATCC 25740 was assessed. It appeared that the liquid substrate in the case of food extracts is the most appropriate since no interference to the diffusion of nisin was detected and the results expressed as percentage of growth inhibition of the indicator organism are very close to those produced by standard nisin solutions allowing this way a direct estimation of the nisin content. The shrimp paste extracts which produced inhibition zones following the treatment for inactivation of nisin, were also examined with the turbidometric assay using the nisin producer *L. lactis *ATCC 11454 as an indicator microorganism. While *L. lactis *appeared to be unaffected by nisin in alkali treated food extracts, which produced no zones by themselves, to which nisin was added in high concentrations, in the case of shrimp paste extracts its growth was severely restricted. Thus, in estimating residual nisin in foods, an easy way to discriminate from other antibiotics prior to assaying for nisin is to perform the turbidometric assay using the nisin producer as indicator. 100% inhibition was produced by 75 IU nisin/ml in both *L. curvatus *and *P. acidilactici*, while a 30% growth inhibition was produced by the addition of nisin at 1 IU/ml in *L. curvatus *(Fig. 9).

## Conclusion

Sensitivity limits and linearity of responses to various bacteriocin levels vary significantly among different test-microorganisms in both bioassays, the lower sensitivity limits depending on both the test-microorganism and the applied method. From the nine test-microorganisms used, only two of them, *L. curvatus *ATCC 51436 and *P. acidilactici *ATCC 25740, were sensitive to very low nisin concentrations and produced a linear type of response in both the plate agar diffusion and turbidometric assays, performed with standard nisin solutions and fermentation broth filtrates. Very low nisin concentrations, e.g. 1 IU/ml, were more safely determined in the turbidometric assay through determination of the percentage of inhibition of growth of the indicator microorganism. This method proved to be more suitable for determination of nisin in processed food samples.

Although the agar diffusion assay is the most widely used method in routine measurements of bacteriocin activity, turbidometry offers a simpler, faster and more reliable alternative since diffusion related problems are eliminated, the degree of human intervention and judgment is low, and very low bacteriocin concentrations can be quantified.

## Materials and methods

### Standard solutions of nisin

These were prepared with "Nisin from *Streptococcus lactis*" from Sigma-Aldrich Fine Chemicals (Cat. Number N5764) which contains 2.5% nisin (balance sodium chloride and denatured milk solids). Nisin standards were prepared by adding 0.1 g nisin to 10 ml 0.02 N HCl and 0.75% NaCl. Sigma-Aldrich does not give the international units equivalent per gram of nisin. However, since the commercial grade standardized Nisaplin^® ^by Aplin and Barrett, as well as, the Nisaplin^® ^by Danisco are produced by *S. lactis *(syn *Lactococcus lactis*) and contain 2.5% nisin and both contain by definition 10^6 ^IU of nisin per gram, we assume the same equivalent for nisin by Sigma-Aldrich, making the working standard of 10.000 IU/ml. Control experiments determined that Nisin from Sigma-Aldrich and Nisaplin^® ^from Danisco give identical results. Steam sterilization was done at 121°C for 15 minutes to ensure activity. Standards were prepared daily. The following nisin concentrations were used, in IU/ml: 1, 2.5, 5, 10, 20, 30, 40, 50, 75, 100, 200, 400, 600, 800, and 1.000.

### Stock cultures of test organisms

Microbial strains were purchased from the Collection Espanola de Cultivos Tipo (CECT) and the American Type Cultures Collection (ATCC). The following strains were used: *Lactobacillus sakei *CECT 906T, *L. plantarum *CECT 220, *L. curvatus *ATCC 51436, *Micrococcus varians *CECT 246, *M. luteus *CECT 241, and *M. flavus *ATCC 400, *Leuconostoc mesenteroides *subsp.*cremoris *ATCC 19254, *Pediococcus pentosaceus *ATCC 33316 and *P. acidilactici *ATCC 25740. For long-term storage, stock cultures were maintained at -80°C in 20% glycerol. Short-term maintenance was done in agar plates of the appropriate substrate at 3°C. Following one subculture, microorganisms were grown in MRS broth (*Pediococcus *spp., *Lactobacillius *spp.), or TGE medium (*Leuconostoc *spp.) [22], or nutrient broth (*Micrococcus *spp.) [16] at 30°C, pH 6.5, for 24 hours. During cultivation, optical density readings were performed at wavelengths ranging from 600 to 700 nm. Maximum absorbance was observed at 650 nm and this wavelength was applied in turbidity measurements (OD_650_).

### Agar diffusion assay

MRS, TGE, or nutrient agar (2%) were used as substrates. Tween 20 was added prior to dispensing the agar into plates. 2% of a 1:1 mixture of Tween 20 and sterile distilled water, previously held for 30 minutes at 48°C, was added and thoroughly mixed with the medium. For the purpose of comparison, assays were carried out without surfactants, or with addition of Tween 80 to agar substrate. The use of surfactants in the agar media, eg. Tween 80 and Tween 20 was previously demonstrated to increase nisin diffusion [17]. Our preliminary tests showed that the use of Tween 20 resulted in maximum assay reproducibility compared to assays performed with Tween 80 or without surfactants (results not shown).

The molten agar was tempered at 40°C, and inoculated with the indicator organism at the middle of exponential phase to a final concentration of 1%. The inoculated medium was poured in sterile Petri dishes and allowed to solidify. Care was taken to ensure addition of a fixed volume of agar to the Petri dishes by using an automatic pipette. After agar solidification, wells of 5 mm diameter were cut with the use of a glass tube. The amount and state of the indicator strain at the inoculation time-point have not been specified in previously published methods. Daba et al. [29] used 50 μl of a 100-fold diluted overnight culture, whereas Mortvedt and Nes [27] used 20 of fresh indicator strain with an OD_600 _between 0.1 and 0.6. In preliminary tests, we examined various combinations of amounts, in the range of 20 to 50 μl, and states of cultures searching for linear-type responses to nisin doses. It appeared that the indicator microorganism concentration strongly influenced the inhibition curves. Having obtained linear responses from two microorganisms at mid-logarithmic phase using the amount of 25 μl, we decided to keep the inoculation amount stable at 25 μl and compare the performances of various test-microorganisms on this base.

25 μl of nisin standard were pipetted into each well and the plates were pre-incubated at 3°C for 24 hrs or were left at ambient temperature for 3 hours, or finally incubated directly at 30°C for 24 hours for comparison. The largest variation and lower sensitivity of all test-microorganisms were obtained when plates were pre-incubated at 3°C for 24 hours. This is directly opposite with the observations of Rogers and Montville[16] who suggested a pre-incubation period of 24 hours at 3°C to ensure maximum assay reproducibility. All tested strains exhibited their maximum sensitivity when pre-incubated at room temperature for 3 hours prior to incubation at 30°C. Direct incubation at 30°C led to a large amount of variation between samples. Therefore, the short pre-incubation period at ambient temperature was incorporated in the standard assay procedure.

Diameters of zones produced in assay plates were measured on images of the plates using an automated image analyzer. Images of the agar plates were taken with a Sony ST-50 video camera and measurements performed using the SIS GmbH software (Soft Imaging System, Olympus, Germany). Background filters and selection based on grayness levels (0–100) were applied in order to include unclear zones in measurements. 10 zones were measured for each nisin level per assay. For oval inhibition zones, the mean of the largest and shortest diameter was calculated. Average zone diameters are presented in this work. Assay experiments were done in triplicates.

### Turbidometric assay

The assay was carried out on culture tubes containing 5 ml of the appropriate liquid media. 100 μl of nisin standard solutions were added to them in concentrations of the same range applied in the PADA. Preliminary tests on assay pH showed that among 4 pHs tested, 5.0, 5.5, 5.8 and 6.0, test-microorganisms responded better -in terms of sensitivity and reproducibility- at pH 5.8 and this was chosen as the assay pH. Studying various aspects of the turbidometric assay for bacteriocin quantification, Cabo et al. [22] observed that indicator microorganism's *L. mesenteroides *responses were better at pH 5.5 or 6, without discriminating between the two. Four different exposure times were also tested. Microorganisms were incubated with nisin for 2, 4, 6 and 8 hours prior to absorbance readings. The variation in incubation time is based on the fact that the slope corresponding to the effect of the bacteriocin will vary throughout the time course of growth, in which case the most discriminatory test would be that with the steepest slope. The incubation time of 6 hours was chosen for the steepest slopes obtained for all test microorganisms (results not shown). Therefore, the pH of 5.8 with an incubation time of 6 hours, were applied as provisional assay conditions.

Culture tubes in which the diluted bacteriocin was substituted by distilled sterile water served as controls. Turbidity measurements were done at 650 nm wavelength. Dose-response curves were obtained from experimental data and the percentage of inhibition of growth of the test-microorganisms was estimated against the control. Assay experiments were done in triplicates.

### Fermentation

Nisin producer *Lactococcus lactis *ATCC 11454 was grown in M17 (Difco) medium, supplemented with 0.5% glucose, in 250 ml Erlenmeyer flasks (50 ml working volume), at 30°C. Stock cultures maintained on M17 agar plates at 3°C. The pH was adjusted at 5.8 following sterilization. Sterilization of medium was done at 121°C for 15 minutes. During fermentation, samples were taken at inoculation time and at 1-hour intervals to 48 hours maximum. *L. lactis *fermentations were done in triplicates.

### Treatment of fermentation samples

The post-incubates of the producer bacteria were buffered with 0.05 M biphthalate-HCl, within the pH range of 3–6.5 (step, 0.5), and centrifuged at 15,000 × g for 10 min, both with and without a prior heating process at 80°C for 3 min. The heating process, made possible by the fact that nisin is thermostable in acidic pHs [23], is applied in order to facilitate extraction and render inactive any proteases that might be present in the broth. The extracts were then tested for nisin by the plate agar diffusion and the turbidometric assays, and the results showed an optimum performance of heat treatment at pH 3. It was therefore decided to add this treatment to the general conditions of the experiment.

### Treatment of food samples

When estimating residual nisin in processed foods, one has to consider the adsorption of nisin on proteins, the heat stability of the peptide at a given pH value, and various substances present in food which may interfere with the assay. In preparation for nisin assay, samples of food were macerated to release nisin from proteins, by acidification with HCl to pH 2, following by boiling for 5 min and centrifugation, in order to bring nisin into the aqueous phase. At a second stage, nisin-containing extracts were made alkaline by adjusting the pH to 11, heated to 65°C for 30 min, causing rapid inactivation of nisin, in order to provide a suitable nisin-free diluent for the nisin standard, and then re-acidified to pH 2. This procedure made possible to use food extracts freed from nisin as controls, by simply incorporating into them certain known concentrations of nisin. The efficiency of the procedure was tested the following way: 50% w/v acid extracts were prepared from cream of chicken soup, shrimp and cheese paste, mayonnaise, canned marinated herrings, and cream cheese, 100 IU/ml of nisin were added to each, and the mixtures made alkaline to pH 11 with 5 N NaOH, incubated for 30 min at 65°C, and re-acidified with 5 N HCl to pH 2. A nisin solution containing 100 IU/ml in 0.02 N HCl was further diluted with either 0.02 N HCl to 50, 20, 10, 5, 2.5, and 1 IU/ml, or with the alkali-treated food extracts. Controls without nisin, consisting of the diluents alone, were also examined. The samples were tested for nisin with the plate agar diffusion and the turbidometric assays. The turbidometric assay was also performed in the case of food extracts using *L. lactis *ATCC 11454 as indicator of the presence of antibiotic substances-other than nisin-in the food samples. Tests were carried out in triplicates.

### Statistical analysis

The effect of the factors X1 = dose, X2 = microorganism, and their interdependence X1*X2 on the variables Y1 = zone diameter (agar diffusion assay) and Y2 = % inhibition of growth (turbidometric assay), as well as pairwise comparisons (Least Significant Difference tests based on the linearly independent pairwise comparisons among the estimated marginal means) of X1 and X2, were examined for the agar diffusion and the turbidometric assays for the following microorganisms: *L. plantarum*, *L. curvatus*, *L. sakei*, *M. varians*, *P. acidilactici *and *P. pentosaceus*. Data were statistically analyzed with the technique of the univariate analysis of variance. The model for the measured response (two factor Nested Design) was applied, which is described by the equation *X*_*ijk *_= *μ*_*ij *_+ *ε*_*k*(*ij*) _= *μ *+ *α*_*i *_+ *β*_*j*(*i*) _+ *ε*_*k*(*ij*)_, where I = 1,2,..., α, j = 1,2,.., β, and k = 1, 2,.., n [30]. Data were analyzed using the software SPSS 12 for Windows.

## Authors' contributions

All authors contributed equally in this work.
